# Development of a new extraction method based on high-intensity ultra-sonication to study RNA regulation of the filamentous cyanobacteria *Planktothrix*

**DOI:** 10.1371/journal.pone.0222029

**Published:** 2019-09-06

**Authors:** Sandra Kim Tiam, Katia Comte, Caroline Dalle, Charlotte Duval, Claire Pancrace, Muriel Gugger, Benjamin Marie, Claude Yéprémian, Cécile Bernard

**Affiliations:** 1 UMR 7245 Molécules de Communication et Adaptations des Microorganismes, Muséum National d’Histoire Naturelle, Paris, France; 2 Collection des Cyanobactéries, Institut Pasteur, Paris, France; University of Helsinki, FINLAND

## Abstract

Efficient RNA extraction methods are needed to study transcript regulation. Such methods must lyse the cell without degrading the genetic material. For cyanobacteria this can be particularly challenging because of the presence of the cyanobacteria cell envelope. The great breath of cyanobacterial shape and size (unicellular, colonial, or filamentous multicellular) created a variety of cell lysis methods. However, there is still a lack of reliable techniques for nucleic acid extraction for several types of cyanobacteria.

Here we designed and tested 15 extraction methods using physical, thermic or chemical stress on the filamentous cyanobacteria *Planktothrix agardhii*. Techniques based on the use of beads, sonication, and heat shock appeared to be too soft to break the *Planktothrix agardhii* cell envelope, whereas techniques based on the use of detergents degraded the cell envelope but also the RNA. Two protocols allowed to successfully obtain good-quality RNA. The first protocol consisted to manually crush the frozen cell pellet with a pestle and the second was based on the use of high-intensity ultra-sonication. When comparing these two, the high-intensity ultra-sonication protocol was less laborious, faster and allowed to extract 3.5 times more RNA compared to the liquid nitrogen pestle protocol. The high-intensity ultra-sonication protocol was then tested on five *Planktothrix* strains, this protocol allowed to obtain >8.5 μg of RNA for approximatively 3.5 × 10^8^ cells. The extracted RNA were characterized by 260/280 and 260/230 ratio > to 2, indicating that the samples were devoid of contaminant, and RNA Quality Number > to 7, meaning that the integrity of RNA was preserved with this extraction method. In conclusion, the method we developed based on high-intensity ultra-sonication proved its efficacy in the extraction of *Planktothrix* RNA and could be helpful for other types of samples.

## 1. Introduction

Cyanobacteria are photosynthetic prokaryotes that played a key role on Earth history as they were responsible for the oxygenation of the atmosphere 2.5 billion years ago. Nowadays, these microorganisms and are still essential in ecosystem functioning as they are at the base of the food chain and contribute to primary production [1]. Moreover, they synthetize a variety of secondary metabolites with antibiotic, antifungal, or antioxidant properties that are of great interest for pharmaceutical and other industrial purposes [2]. Despite all these remarkable properties, cyanobacteria are also a source of concern because they can form blooms (i.e., sudden increases of cyanobacteria cellular concentration in water bodies) that modify the dynamics of ecosystems. Such blooms can have sanitary consequences because some cyanobacteria have the capacity to synthetize toxic compounds with deleterious effects on animal and human life [3]. Consequently, cyanobacteria have been intensively studied in diverse areas of research, such as biotechnology, pharmacology, ecology, and ecotoxicology.

Researchers have particularly focused on the genus *Planktothrix* because of the recurrent toxicity of *Planktothrix* bloom [4]. Some *Planktothrix* populations synthesize microcystins that are cyclic heptapeptides and potent cyanotoxins. Microcystins act as hepatotoxins, inhibiting the protein phosphatase type 1 and type 2A [5]. *Planktothrix* genomes have been extensively studied [6–9]. However, few transcriptomic studies documented the regulation of the genetic response of *Planktothrix*. These studies have focused on the regulation of genes belonging to the microcystin gene cluster [10,11] and the Heat Shock Protein family [12]. Hence studies considering a broader set of genes are still needed to better understand the metabolism of *Planktothrix*.

Fluorescence-based quantitative real-time polymerase chain reaction (qPCR) is considered as the gold standard for the measurement of transcript abundance. Nucleic acid extraction is a critical step to ensure reliable measurement of transcript abundance [13]. Nucleic acid extraction should permit the lysing of cells without degrading the genetic material. Such extraction can be particularly challenging when working with Gram negative-type cell envelopes composed of an outer membrane, peptiloglycan layer, and cytoplasmic membrane [14]. The presence of the cyanobacterial cell envelope can make it particularly difficult to lyse the cells. Moreover, the great breath of cyanobacterial shape and size (unicellular, colonial, or filamentous multicellular) created a variety of cell lysis methods using physical, thermic, or enzymatic stress, which were developed in the past [8,10,11,15–25]. However, there is still a lack of reliable techniques for nucleic acid extraction for several types of cyanobacteria.

The objective of this study was to develop a reliable RNA extraction method adapted to *Planktothrix*. Therefore, 15 extraction methods were designed based on the existing homogenization methods for cyanobacteria nucleic acid extraction, including *Planktothrix*. Each of these 15 extraction methods was then tested on *P*. *agardhii* (Gomont) Anagnostidis et Komárek 1988. *Planktothrix agardhii* (S1 Fig) belongs to the order of the Oscillatoriales, which are filamentous cyanobateria with no heterocytes and no akinetes. Like other cyanobacteria, the cell envelope is composed of an outer membrane, peptiloglycan layer, and cytoplasmic membrane. *Planktothrix agardhii* does not have mucilaginous envelopes or sheaths. Trichomes (i.e., a filamentous row of cells) are up to 300 μm long and (2.3) 4–6 (9.8) μm wide (S1 Fig). After extraction by the different methods, the nucleic acid was quantified in the extract to evaluate the ability of the methods to release RNA. Finally, an optimized protocol for RNA extraction for *Planktothrix* was proposed and the suitability for qPCR analyses of the extracted RNA from this optimized protocol was evaluated.

## 2. Material and methods

### 2.1 Cultures of *Planktothrix agardhii*

Cultures of *P*. *agardhii* PCC (Pasteur Culture Collection of Cyanobacteria) 7805 were grown in batches in sterile BG11 medium [26] in 2 L Erlenmeyer flasks. The cultures were maintained in a thermostatic room at 18 °C with a photon flux density of 6 μmol/m^2^/s and a 13:11 h light:dark cycle. It was subcultured every four weeks to promote optimal growth of the cyanobacteria. A volume of 50 mL with an optical density measured at 750 nm (OD_750nm_) of approximately 0.3, which corresponded to approximatively 3.5 × 10^8^ cells (S2 Fig), was sampled and centrifuged at 3220 *g* for 10 minutes at 4 °C. The cell pellets were transferred into 2 mL eppendorf tubes and centrifuged at 3220 *g* for 10 minutes at 4 °C. The pellets were freeze dried under liquid nitrogen and stored at -80 °C until use.

### 2.2 RNA extractions protocols

**Fig 1:**
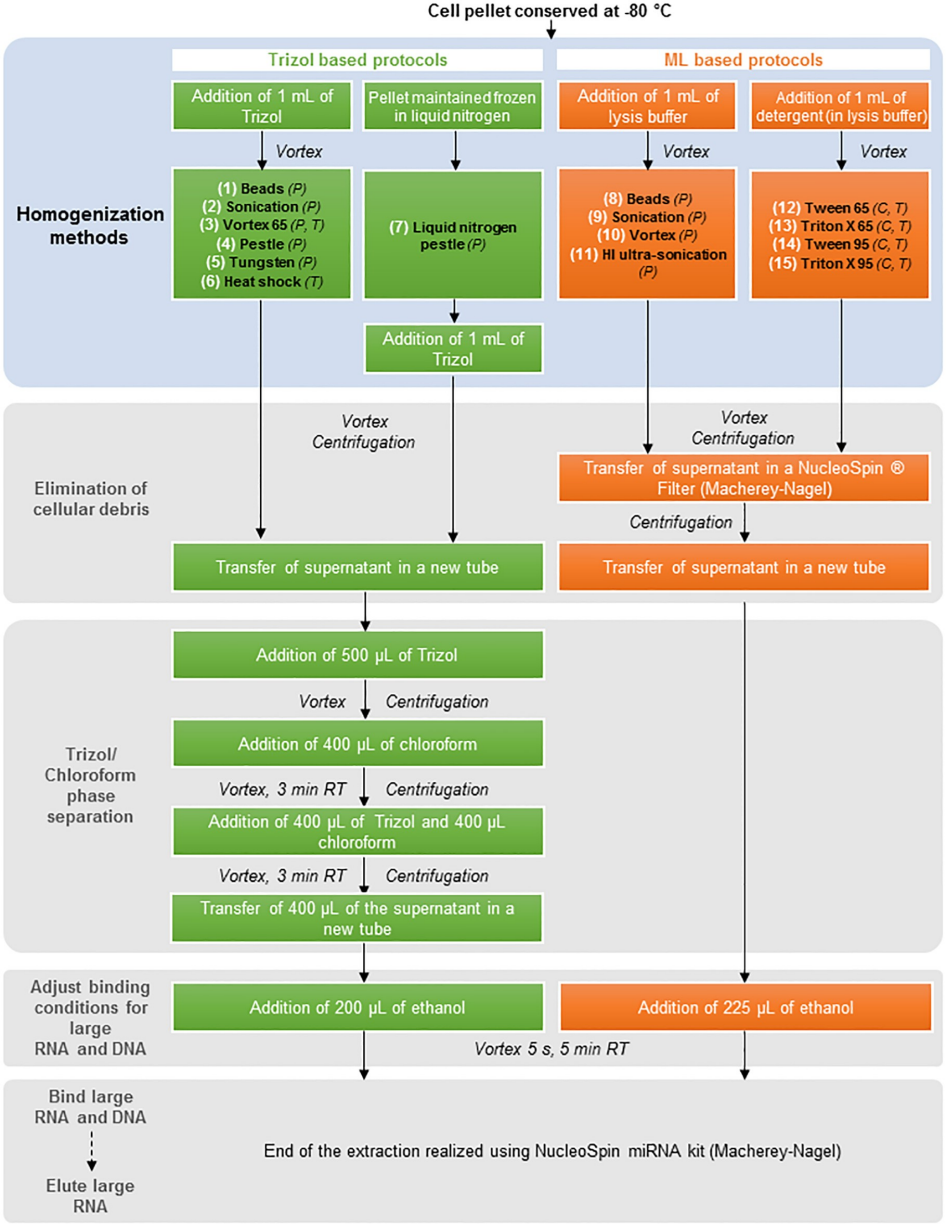
Protocols designed in this study in order to extract *Planktothrix agardhii* RNA. These protocols numbered from (1) to (15) differed in terms of the extraction solution used and the homogenization method applied. P: physical stress; T: thermic stress, and C: chemical stress.

The bibliographic review (Table 1) revealed few but diverse RNA extraction protocols, based on which we designed several protocols with tests numbered from (1) to (15) (Fig 1). These protocols differed in terms of the extraction solution used and the homogenization method applied. Regarding the extraction solution, the protocols tested could be divided into two categories: Trizol (Invitrogen)-based (1–7) and Lysis buffer ML-based (Macherey-Nagel) (8–15) protocols. Trizol is a commercial monophasic solution of phenol and guanidine isothiocyanate that maintains the integrity of the RNA because of a highly effective inhibition of RNase activity while disrupting cells and dissolving cell components during sample homogenization. Trizol is an improvement to the single-step RNA isolation method originally based on acid guanidinium thiocyanate–phenol–chloroform extraction (Chomczynski and Sacchi, 1987). Alternatively, commercially available extraction kits from Macherey-Nagel propose to isolate RNA without the need of cumbersome phenol/chloroform extraction replacing Trizol by a Lysis Buffer (ML). Concerning homogenization, physical stress (P), thermic stress (T), chemical stress (C) and a combination of several stresses were applied.

**Table 1:** Main existing extraction protocols for cyanobacteria nucleic acid. Biological model, homogenization method, and extraction solution used in these studies are documented.

Reference	Biological model	RNA/DNA	Homogenization method			Extraction solution
			Physical	Thermic	Enzymatic	
*Kaebernick et al*. *2000*	*Microcystis aeruginosa* PCC 7806	RNA	**pestle and mortar**	**_**	**_**	Trizol (cells powder transfered into)
*Tillett and Neilan 2000*	*M*. *aeruginosa* PCC 7806, *M*. *wesenbergii* NIES 107, *M*. *viridis* NIES 102, *M*. *incerta* HINDAK 1965/17, *Nodularia spumigena* PCC 73104, *Pseudanabaena* sp. AWT 210 and *Anabaena circinalis* AWT 006	RNA	**_**	**65°C**	**_**	XSD (xanthogenate-SDS-phenol)
*Dittmann et al*. *2001*	*Microcystis aeruginosa* PCC 7806	RNA	**pestle and mortar**	**_**	**_**	Trizol (cells powder transfered into)
*Tonk et al*. *2005*	*Planktothrix agardhii* 126/3	RNA	**pestle and mortar**	**_**	**_**	Trizol (cells powder transfered into)
*Kim et al*. *2006*	*Merismopedia tenuissima* NIES 230, *Synechococcus leopoliensis* UTEX 625, *Arthrospira platensis* NIES 39, *Oscillatoria tenuis* NIES 33, *Phormidium* sp. KCTC AG10164, *Synechocystis* sp. PCC 6803, *Anabaena flos-aquae* UTEX 2557, *Nodularia spumigena* UTEX 2092, *Nostoc* sp. PCC 7120, *Microcystis aeruginosa* UTEX 2666	RNA	**bead beating**	**_**	**_**	deionized water and saturated phenol
*Pinto et al*. *2009*	*Nostoc punctiforme* ATCC 29133	RNA	**_**	**95°C**	**_**	PGTX (phenol, glycerol, guanidine and triton X)
			**_**	**95°C**	**_**	Trizol
			**_**	**_**	**_**	Trizol
			**bead beating**	**_**	**_**	PGTX
			**bead beating**	**_**	**_**	Trizol
			**bead beating**	**_**	**_**	deionized water and saturated phenol
*Singh et al*. *2010*	*Anabaena variabilis* PCC 7937	RNA	**_**	**freezing/ thawing**	**_**	Trizol
*Sipari et al*. *2010*	*Anabaena* sp. 90 and *Microcystis aeruginosa* PCC 7806	RNA	**bead beating**	**_**	**_**	commercial lysis buffer (PMR1, Mo Bio)
*Churro et al*. *2012*	*Planktothrix agardhii* LMECYA 153B	DNA	**sonication and beads beating**	**_**	**_**	not specified
*Pinto et al*. *2012*	*Synechocystis* sp. PCC 6803, *Lyngbya aestuarii* CCY 9616 and *Nostoc* sp. PCC 7120	RNA	**bead beating**	**_**	**_**	Trizol
*Tran 2012*	*Planktothrix agardhii* CYA126/8	RNA	**_**	**95°C**	**_**	Trizol
*Penn at al*. *2014*	surface water collected from freshwater reservoir	RNA	**bead beating**	**_**	**lysozyme**	Trizol (cells transfered after enzymatic digestion)
*Salvador et al*. *2016*	*Planktothrix agardhii* LMECYA 256 and *Microcystis aeruginosa* LMECYA 7	RNA	**bead beating**	**freezing/ thawing**	**_**	Trizol

In this study, we designed different RNA extraction protocols based on the homogenization methods presented in Table 1. In the following sections, all centrifugations were carried out at 10,000 x *g*. Centrifugation was done for 10 minutes at 4 °C unless specified.

#### 2.2.1 Trizol protocol-based RNA extractions

1 mL of Trizol (Invitrogen) was added to the freeze dried pellet and the tube was vortexed. In the “Beads” protocol (1), the pellet was resuspended in Trizol and was transferred into a clean eppendorf tube containing 300 μL of glass beads (0.1mm diameter). Further, the sample was vortexed three times for 30 s. In the “Sonication” protocol (2), the sample was subjected to 20 min of sonication in an Elmasonic S10-H instrument (Elma^®^). For the protocol “Vortex 65” (3), the sample was incubated at 65 °C for 12 min, vortexed three times during the incubation period and then centrifuged. For the protocol “Pestle” (4), the pellet was crushed manually with a single-use pestle (Argos Technologies) for approximatively 30 s. In the “Tungsten” protocol (5), the pellet was resuspended in Trizol and transferred into a clean eppendorf tube containing two tungsten carbide beads (3 mm Qiagen). Then, the sample was processed three times for 30 s at 30 kHz with the TissueLyser system (Qiagen). In the protocol “Heat shock” (6), the tube was immerged in liquid nitrogen and then incubated at 95 °C for 5 min; the operation was repeated two times. For the protocol “Liquid nitrogen pestle” (7), the frozen pellet was crushed manually with a single-use pestle three times for 30 s, with the immersion of the tube in liquid nitrogen between each cycle, and then 1 mL of Trizol was added.

After cell lysis, the sample was vortexed and then centrifuged to allow cellular debris to settle. The supernatant was transferred into a clean eppendorf tube and 500 μL of Trizol was added; the tube was vortexed for 30 s and then centrifuged. A volume of 400 μL of chloroform (Sigma) was added, the sample was vortexed, incubated for 3 min at room temperature, and was then centrifuged. Volumes of 400 μL of Trizol and 400 μL of chloroform were added and the sample was vortexed. After 3 min of incubation at room temperature, the sample was centrifuged, and the supernatant was transferred into a clean eppendorf tube. After the addition of 200 μL of ethanol, the tube was vortexed for 5 s and incubated 5 min at room temperature. The end of the extraction was realized using a NucleoSpin miRNA kit (Macherey Nagel) according to the manufacturer’s instructions. The large RNA fraction (>200 nucleotides) was recovered in 50 μL RNAse/DNAse free sterile water according to the instructions in the protocol section “Small and large RNA in two fractions”.

#### 2.2.2 Lysis buffer ML protocol-based RNA extractions

A volume of 1 mL of lysis buffer (Lysis Buffer ML, Macherey-Nagel) was added to the frozen pellets and the tube was vortexed. In the “Beads” protocol (8), the pellet was homogenized in the lysis buffer and transferred into a clean eppendorf tube containing 300 μL of glass beads (0.100 mm diameter). Then, the sample was vortexed three times for 30 s each. In the “Sonication” protocol (9), the sample was subjected to 20 min of sonication in an ultrasonic bath. For the protocol “Vortex” (10), the sample was incubated at room temperature for 12 min and vortexed three times during the incubation time. For the “HI ultra-sonication” (11), the sample was placed in a cup horn (High Intensity Cup Horns, SONICS) filled with autoclaved H_2_O Milli-Q at 4 °C and submitted to High Intensity (HI) ultra-sonication with an ultrasonic Processor (VCX 130, SONICS) two times for 30 s each at 20% of maximal power. The sample was vortexed after each 30 s pulse.

A volume of 1 mL of Tween 20 (0.2% in ML lysis buffer) or 1 mL of Triton X (0.2% in ML lysis buffer) was added to the frozen pellets and the tube was vortexed. Then, the sample was incubated for 30 min at 65 °C (for the protocols “Tween 65” (12) and “Triton 65” (13)) or at 95 °C (for “Tween 95” (14) and “Triton 95” (15)). The sample was vortexed three times during the incubation time.

After cell lysis, the sample was vortexed and then centrifuged to allow the cellular debris to settle. The supernatant was transferred into a NucleoSpin Filter and centrifuged to eliminate the remaining cellular debris. Because the pellet formation was sometimes observed in the collection tube, 600 μL of supernatant was transferred into a new tube taking care not to disturb the pellet. After the addition of 225 μL ethanol, the tube was vortexed for 5 s and incubated 5 min at room temperature.

The end of the extraction was realized using the NucleoSpin miRNA kit (Macherey Nagel) according to the manufacturer’s instructions. The large RNA fraction (>200 nucleotides) was recovered in 50 μL RNAse/DNAse free sterile water according to the instructions in the protocol section “Small and large RNA in two fractions”.

RNA integrity was analyzed on 1% (w/v) agarose gel with SYBR^™^ Green I Nucleic Acid Gel Stain (Invitrogen) by UV light on an illuminator. To confirm RNA integrity, the samples were analyzed using RNA Nano chips on a Bioanalyzer (Agilent Technologies). RNA quantity and purity was assessed using a Nanodrop Spectrophotometer (ThermoFisher Scientific).

### 2.3 Reverse transcription of RNA

The first strand of cDNA was synthesized from 1 μg of RNA in a final volume of 20 μL using a High-Capacity cDNA Reverse Transcription Kit (ThermoFisher Scientific) according to manufacturer’s instructions (one step at 25 °C for 10 min, one step at 37 °C for 120 min, and one step at 85 °C for 5 min). Reverse Transcription was realized in a GeneTouch Thermal Cycler (BIOER Technology) and the cDNA was stored at -20 °C until it was used in a qPCR reaction. The MultiScribe^™^ Reverse Transcriptase was replaced by PCR-grade water for non-retro transcript samples.

### 2.4 qPCR primer design and qPCR parameters

The DNA gyrase subunit B (*gyrB*), the ribonuclease P protein component (*rnpA*), and the 30S ribosomal protein S12 (*rpsL)* were the three genes selected to evaluate the suitability of the extraction technic for qPCR analyses. Primer pairs (Table 2) of 20 bp long were designed for amplification of fragments between 198 and 215 bp using he NCBI primer-Blast tool.

**Table 2:** Primer pairs tested for the three *Planktothrix agardhii* PCC 7805 genes in qPCR realized with the RNA obtained from the RNA extraction protocol developed in this study.

Gene name	Gene abbreviation	Primer names	Primers sequence (5'-3')	Amplicon size (bp)
DNA gyrase subunit B	*gyrB*	gyrB_S	AAATGCGATCGCCCGTAAAC^a^	198
		gyrB_AS	TCGGTTAAAGCTTCCCCCAC^b^	
30S ribosomal protein S12	*rspL*	rpsL_S	AAAATCGCCCGCCCTAAAGA^a^	215
		rpsL_AS	GCAAATCTTTCACCCGACCG^b^	
DNA-directed RNA polymerase subunit gamma	*rpoC*	rpoC_S	TATCCCCAGCAACGGGTAGA^a^	198
		rpoC_AS	TCTCCCCATCAAATCGCACC^b^	

^a^ Forward primer;

^b^ Reverse primer.

The qPCRs were performed in the LightCycler 480 (Roche) following the manufacturer’s instructions (one cycle at 95 °C for 5 min and 45 amplification cycles at 95 °C for 10 s, 60 °C for 10 s, and 72°C for 10 s). Each 20 μL reaction contained 3 μL of PCR-grade water (Roche), 2 μL of the gene-specific primer pair at a final concentration of 300 nM for each primer, 10 μL of MasterMix (Roche), and 5 μL of cDNA. For non-template controls (NTC), cDNA was replaced by PCR-grade water. For non-retro transcript controls (NRT), the cDNA was replaced by non-retro transcript RNA. Each qPCR was realized in duplicate.

#### 2.4.1 Primer specificity

Specificity was checked for each reaction by analyzing the PCR product using melt curve analysis, gel analysis, and sequencing. The dissociation curve of the PCR product was obtained by following the SYBR Green fluorescence level during gradual heating of the PCR products from 65 to 95 °C. In addition, samples were run on 2% (w/v) agarose gel to confirm that the amplicon was of the expected size. Successful amplifications were sent for sequencing and then blasted on the Microbial Genome Annotation and Analysis Platform (http://www.genoscope.cns.fr/agc/microscope).

#### 2.4.2 PCR efficiency

A standard curve was used to determine the reaction efficiency by producing a 10-fold dilution series over five points from the most concentrated sample. The qPCR reaction was performed for the three primer pairs used in the experiment. LC480 Software (Roche) was used to construct the standard curves by plotting the log of the starting quantity of the template against the C_t_ values obtained. The PCR efficiency (E) is given by the equation: E = 10 ^(-1/slope)^ − 1. The theoretical maximum of 1 (or 100%) indicates that the amount of product doubles with each cycle.

## 3. Results and discussion

### 3.1 Tested RNA extraction protocols

Homogenization methods for cyanobacterial nucleic acid extraction have been developed for cyanobacteria, including *Planktothrix agardhii* (Table 1). These methods used physical, thermic, or enzymatic stresses or a combination thereof to lyse cyanobacterial cells. Physical stresses included the use of pestle and mortar to crush the frozen sample [11,16,17], bead beating [10,15,18–21,23], or sonication [15]. Thermic stress has been applied by repeated freezing/thawing processes [10,22] in which samples were rapidly frozen leading to the formation of ice crystals in the cells and the rupture of membranes. In other cases, samples have been incubated at high temperature [21,25,27] to increase reaction kinetics with the membrane disrupting component chemicals contained in the extraction buffer. Homogenization methods also include the use of enzymes as lysozymes [19] that digest the cell envelope components. Table 1 illustrates the diversity of homogenization methods that can be used for cyanobacteria. This table will be helpful for future studies dealing with cyanobacteria nucleic acid extraction because information in numerous papers is now synthetized here.

RNA quantification and integrity for the different extraction methods tested for *P*. *agardhii* PCC 7805 are resumed in Table 3.

**Table 3:** Mean RNA concentration (± standard deviation; n = 2) for the different homogenization methods tested for *Planktothrix agardhii* PCC 7805. RNA integrity (non degraded/degraded) is indicated. *for 50 mL of culture at an OD_750nm_ = 0.3 recovered in 50 μL RNAse/DNAse free sterile water; n.a.: non-available. Detection limit (d.l.) was 1.6ng/μL.

*Extraction solution*	Homogenization method	RNA concentration (ng/μL)*	*RNA integrity (electrophoresis)*
***Trizol based protocols***	***(1) Beads***	<d.l.	n.a.
	***(2) Sonication***	<d.l.	n.a.
	***(3) Vortex 65***	<d.l.	n.a.
	***(4) Pestle***	<d.l.	n.a.
	***(5) Tungsten***	<d.l.	n.a.
	***(6) Heat shock***	<d.l.	n.a.
	***(7) Liquid nitrogen pestle***	**34.1 ± 4.6**	**non degraded**
***ML based protocol***	***(8) Beads***	<d.l.	n.a.
	***(9) Sonication***	<d.l.	n.a.
	***(10) Vortex***	<d.l.	n.a.
	***(11) HI ultra-sonication***	**120.8 ± 11.8**	**non degraded**
	***(12) Tween 65***	37.7 ± 7.6	degraded
	***(13) Triton X 65***	39.5 ± 23.1	degraded
	***(14) Tween 95***	83.8 ± 1.3	degraded
	***(15) Triton X 95***	75.9 ± 9.8	degraded

**Fig 2:**
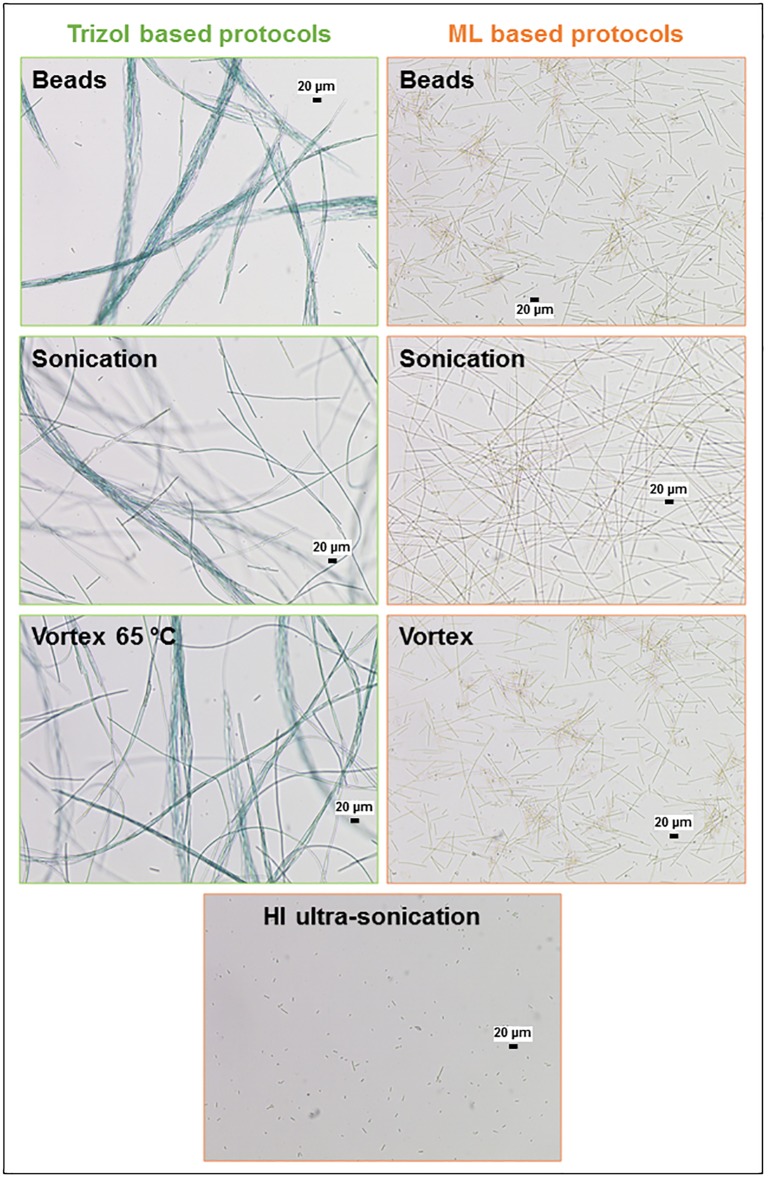
Microscopic observations (n = 2) of *P*. *agardhii* after “Beads,” “Sonication,” “Vortex 65,” and “HI ultra-sonication” RNA extraction protocols realized in Trizol or Lysis buffer ML.

RNA was not detected for the “Beads” (1), “Sonication” (2), “Vortex 65” (3), “Pestle” (4), “Tungsten” (5), or “Heat shock” (6) of the Trizol-based protocols, nor for the “Beads” (8), “Sonication” (9), or “Vortex” (10) of the Lysis Buffer ML-based protocols. To identify if the unsuccessful step was cell lysis or the separation of the RNA from cellular debris, samples were observed under a microscope after the homogenization step (Fig 2). After the Trizol-based extractions, intact trichomes of *Planktothrix* were aggregated. After Lysis Buffer ML-based protocols, the trichomes also remained intact, but without aggregation and some trichomes were even broken (short fragments of trichomes present). Nevertheless, RNA concentrations were below the detection limit (Table 3) revealing that RNA was not released into the extraction buffer.

Hence, these techniques were unable to break the cellular envelop of *Planktothrix*, despite the use of high temperature, bead beating, and sonication already successful for *Planktothrix agardhii* cell lysis [10,15,25]. The impairment of *Planktothrix agardhii*, a genus yet without mucilaginous envelopes or sheaths, cell lysis in our study could be linked to cell envelop thickness, strain specific-characteristics, or by culture conditions. Moreover, we observed that the addition of Trizol or lysis buffer ML to the frozen pellet led to the formation of highly viscous samples preventing efficient sample homogenization. In the mechanical protocols using glass, tungsten beads, or a pestle, the beads/pestle performed poorly in terms of breaking *Planktothrix* cells and trichomes. Additionally, extraction methods based on the use of detergents were tested (12 to 15) with nonionic detergents used to solubilize protein membranes, such as Tween 20 and Triton X. These protocols effectively denatured the cell envelope because nucleic acids could be quantified but resulted in low-quality RNA as revealed by the results of electrophoresis analyses (Table 3, S3 Fig). Consequently, protocols based on the use of detergents were also excluded.

In the protocol “Liquid nitrogen pestle” (7), the frozen pellet was crushed with a pestle and the tube was immerged several times in liquid nitrogen between each crushing of the cells to ensure that the sample was still in a frozen state. Electrophoresis (S3 Fig) revealed the conservation of RNA with a concentration of 34.1 ± 4.6 ng/μL. This protocol successfully obtained good-quality RNA but was difficult to apply for a large number of samples because of its laborious and time-consuming nature. Moreover, the quantity of RNA extracted was relatively low.

The “HI ultra-sonication” treatment (11) resulted in high-quality RNA (S3 Fig), at a concentration superior to 120.8 ± 11.8 ng/μL (Table 3). The samples were analyzed using RNA Nano chips on a Bioanalyzer (Agilent Technologies) to confirm RNA integrity, the RNA Quality Number (RQN) was closed to seven indicated good-quality samples (S4 Fig). In ultra-sonication, vibrations are transmitted into the liquid as alternating expansive and compressive acoustic pressure waves. The pressure fluctuations create millions of microbubbles (cavities), which expand during the low-pressure phases and implode violently during the high-pressure phases (SONICS). The cumulative amount of energy generated by the imploding cavities is extremely high and many times that generated in an ultrasonic bath. Hence, ultra-sonication proved to be particularly efficient for *Planktothrix* cell lysis.

In our study, the *Planktothrix* cell envelope appeared to be particularly difficult to break. Techniques based on the use of beads (1, 3, 5, 8, and 10) sonication (2 and 9), and heat shock (6) appeared to be too soft to break the *Planktothrix* cell envelope, whereas techniques based on the use of detergents (12–15) degraded the cell envelope but also the RNA. Hence, particularly strong techniques were required to lyse the cells without degrading the genetic material. In conclusion, of the 15 RNA extraction protocols tested for *Planktothrix*, only two allowed for the extraction of high-quality RNA: the “Liquid nitrogen pestle” and the “HI ultra-sonication.” When comparing these two, the “Liquid nitrogen pestle” presented some disadvantages linked to the manual nature of cell lysis that did not occur in the “HI ultra-sonication.” Consequently, the “HI ultra-sonication” protocol was selected and further optimized for *Planktothrix agardhii* RNA extraction.

### 3.2 Proposed protocol for RNA extraction

**Fig 3:**
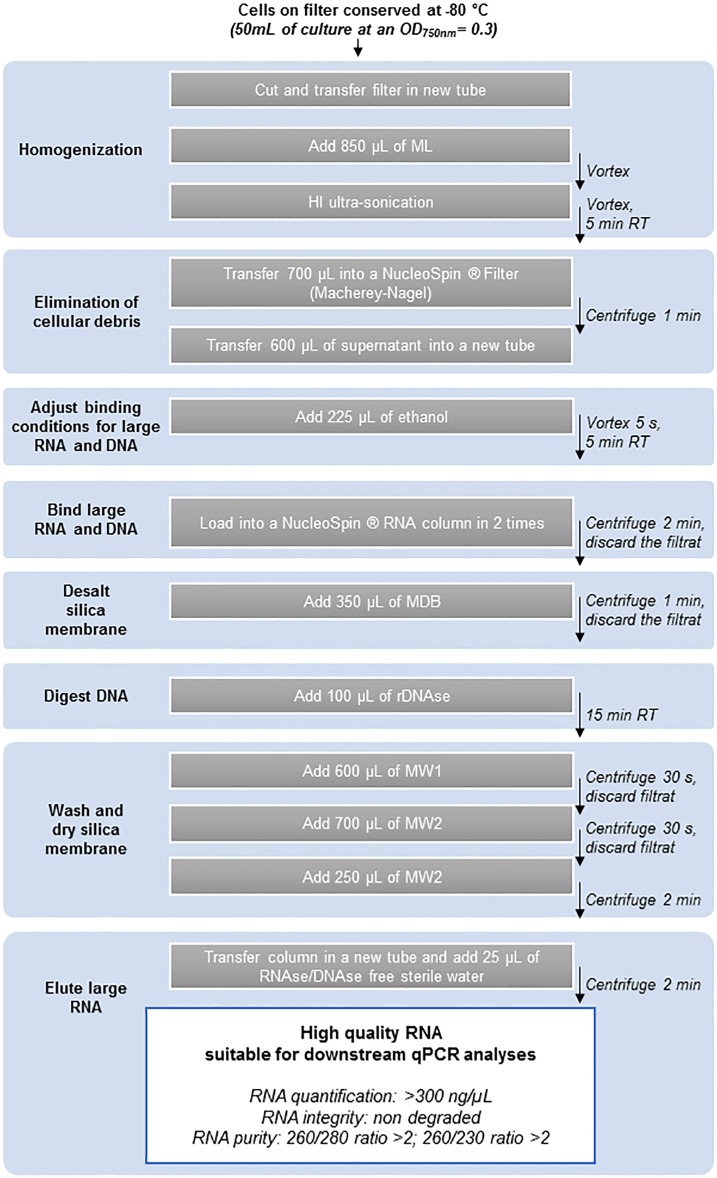
Optimized RNA extraction procedure for *P*. *agardhii*. RNA quantification (Nanodrop), integrity (electrophoresis), and purity (Nanodrop) obtained with the extraction method given for 50 mL of culture at an OD_750nm_ = 0.3 recovered in 25 μL RNAse/DNAse free sterile water.

The proposed protocol for RNA extraction based on the use of HI ultra-sonication is presented in Fig 3. The sampling method was changed from centrifugation to filtration to reduce the duration between sampling and cell storage and consequently minimize the alteration of transcript expression by the sampling technique.

The 50 mL of culture was filtered through a polycarbonate membrane (diam. 47 mm, pore size 3.0 μm Whatman Nucleopore). The filter was transferred into a 2 mL eppendorf tube containing 1.5 mL of RNAlater (Sigma). The tube was stored at 4 °C overnight and then transferred at -80 °C until RNA extraction. After thawing at room temperature, the filters were transferred to a new 2 mL eppendorf tube, cut in small pieces, and 850 μL ML was added. The tube was briefly vortexed and submitted to HI ultra-sonication with an ultrasonic Processor (VCX 130, SONICS) two times for 30 s each at 20% of the maximal power. The sample was vortexed after each 30 s pulse. After 5 min of incubation at room temperature, 700 μL of sample was transferred into a NucleoSpin Filter and centrifuged for 1 min. To eliminate all cellular debris that may affect RNA extraction, 600 μL of the supernatant was transferred into a new tube taking care not to disturb the pellet. A volume of 225 μL of ethanol was added, the sample was vortexed 5 s, and incubated 5 min at room temperature. The large RNA fraction (>200 nucleotides) was recovered in 25 μL RNAse/DNAse free sterile water according to the instructions in the protocol section “Small and large RNA in two fractions”.

This protocol has been tested on five *Planktothrix* strains: *P*. *agardhii* PCC 7805, *P*. *rubescens* PCC 7821, *P*. *agardhiii* PCC 10110, *P*. *agardhii* NIVA CYA 126/8, and *P*. *agardhii* NIVA CYA 126/8 *ΔmcyD*. The RNA concentration, purity and integrity obtained with this protocol for the five strains are presented in Table 4.

**Table 4:** RNA quantification, purity and integrity for the optimized homogenization method based on HI ultra-sonication presented in Fig 3 tested on different *Planktothrix* stains (n = 63 for each strain). RQN: RNA Quality Number.

Strain	RNA quantification* (ng/μL)	RNA purity		RNA integrity
		260/280 ratio	260/230 ratio	RQN
*P*. *agardhii* PCC 7805	357.2 ± 16.5	2.17 ± 0.01	2.30 ± 0.06	8.01 ± 0.13
*P*. *rubescens* PCC 7821	382.8 ± 17.9	2.17 ± 0.01	2.34 ± 0.05	7.65 ± 0.07
*P*. *agardhii* PCC 10110	323.6 ± 12.7	2.18 ± 0.01	2.34 ± 0.05	8.73 ± 0.09
*P*. *agardhii* NIVA CYA 126/8	305.6 ± 12.1	2.17 ± 0.01	2.29 ± 0.08	8.83 ± 0.06
*P*. *agardhii* NIVA CYA 126/8 *ΔmcyD*	344.1 ± 13.2	2.15 ± 0.01	2.30 ± 0.05	9.24 ± 0.05

*for 50 mL of culture at an OD_750nm_ = 0.3 recovered in 25 μL RNAse/DNAse free sterile water.

The use of the HI ultrasonication permitted to extraction RNA at concentrations superior to 300 ng/μL. Moreover, our results indicated that this protocol resulted in high-quality RNA (i.e. RNA purity and RNA integrity). RNA purity was evaluated regarding the 260/280 and the 260/230 absorbance ratio. A 260/280 ratio of ~2.0 is generally accepted as “pure” for RNA. This ratio provides an indication of RNA purity, because the presence of protein, phenol or other contaminants that absorb strongly at or near 280 nm alters the ratio. The 260/230 ratio is used as a secondary measure of nucleic acid purity. Generally accepted 260/230 ratio for good quality RNA are > 2.0–2.2. If the ratio is appreciably lower than expected, it may indicate the presence of contaminants which absorb at 230 nm like EDTA, carbohydrates, phenol or guanidine isothiocyanate. In our study, the mean 260/280 and 260/230 ratio measured for all five *Planktothrix* strains tested were > to 2 indicating that the samples are devoid of contaminant. The integrity of RNA was evaluated with the RNA Quality Number (RQN), this number varies from 0 (totally degraded RNA) to 10 (intact RNA). A RQN value > to 7 is characteristic of samples of high quality. The mean RQN for the samples extracted with the HI ultra-sonication method were > to 7 for all five strains, meaning that the integrity of RNA was preserved with this extraction method.

The new method based on the use of HI ultra-sonication we proposed is rapid and easy to perform and allows for the extraction of RNA with high integrity and high purity, requisite criteria for downstream applications as qPCR.

### 3.3 Suitability of the extracted RNA for qPCR analyses

The suitability for qPCR analyses of the extract, obtained with the optimized protocol for RNA extraction based on HI ultra-sonication, was experimentally assessed regarding PCR efficiency and the coefficient of determination (*r*^2^) reported in Table 5.

**Table 5:** qPCR related parameters for three genes of *Planktothrix agardhii* PCC 7805 studied. The Ct values for the first dilution (d_1_) of the standard curve are given.

Organism	Gene	Amplicon T_m_ (°C)	Ct (d_1_)	*r*^2^	Efficiency (%)	Slope
*Planktothrix agardhii* **PCC 7805**	*gyrB*	81.18	22.45	0.998	104.5	-3.218
	*rpsL*	84.50	20.00	0.998	82.9	-3.812
	*rpoC*	79.08	21.45	0.966	104.3	-3.224

In a perfect qPCR assay, the number of products doubles with each cycle corresponding to the efficiency of 100%. In practical cases, assays with efficiency in the range 80–120% are generally considered acceptable. PCR efficiency out of this range indicates problems with the PCR that can cause artefactual results [28]. Moreover, it is possible to correct the errors in relative quantification because of different efficiencies between the target and reference genes using equations that take efficiency into account [29]. In our study, the three primer pairs tested permitted the realization of qPCR with efficiencies between 82–105% indicating the robustness of the assay. The *r*^2^ value of a standard curve represents how well the model fits the experiment data, a value > 0.980 is desirable for qPCR [28]. In our assay, the value of *r*^2^ met these criteria for most of the standard curves and was always > 0.966.

To conclude, the assessment of assay performance based on the PCR efficiency and the coefficient of determination (*r*^2^) permitted us to validate the primers designed, as well as the suitability of the RNA obtained by the extraction method developed to study the genetic expression of *Planktothrix agardhii* PCC 7805.

## 4. Conclusions and perspectives

Efficient RNA extraction methods are needed to study transcript regulation. The great breath of cyanobacterial shapes and sizes resulted in the variety of cell lysis methods developed in the past years. The information regarding these cyanobacteria cell lysis methods now synthetized in this paper could be helpful for researchers that will want to work on the study of cyanobacteria RNA.

In this work, we showed that accessing the nucleic acids of cyanobacteria could be challenging since most of the protocols we designed, based on existing cyanobacteria cell lysis methods, were too soft to break the *Planktothrix* cell envelope or degraded the RNA. Two methods allowed for the extraction of high-quality RNA, but we showed that using HI ultra-sonication was faster, less laborious, more efficient and applicable to a higher number of samples compared to the liquid nitrogen pestle method.

The new method we proposed based on the use of HI ultra-sonication is rapid, easy, and allows for the extraction of RNA with high integrity and high purity for use for downstream applications, such as qPCR. Moreover, this method could be efficient for a large range of cyanobacteria and of particular interest in the case of samples refractory to disruption.

## Supporting information

S1 Fig *Planktothrix agardhii* PCC 7805 observed (a) under light microscopy and (b,c) under transmission electron microscopy. The red, white, and black arrows point at the peptiloglycan layer, the outer membrane, and cytoplasmic membrane, respectively. Photos: Chakib Djediat.
(TIF)

S2 Fig Cellular concentration (cells/mL) in *P*. *agardhii* samples with an OD_750nm_. of 0.3. The solid line represents the mean cellular concentration (n = 18), (± SD; dashed lines).
(TIF)

S3 Fig RNA integrity assessed by electrophoresis. (a) 1: RNA ladder; 2–3: Tween 95; 4–5: Triton X 95; 6–7: Tween 65; 8–9: Triton X and (b) 1: RNA ladder; 2–3: Liquid nitrogen pestle; 5–6: HI ultra-sonication.
(TIF)

S4 Fig (a) Electropherograms and (b) gel-like images realized with the Bioanalyzer (Agilent Technologies) of *P*. *agardhii* RNA extracted with the “Liquid nitrogen Pestle” or the “HI ultra-sonication” methods. (TIF)
