# Supplementary figures and images for: Development of a new extraction method based on high-intensity ultra-sonication to study RNA regulation of the filamentous cyanobacteria Planktothrix
Source: PLoS One. 2019 Sep 6;14(9):e0222029. doi: 10.1371/journal.pone.0222029 (PMC6730872; doi:10.1371/journal.pone.0222029)

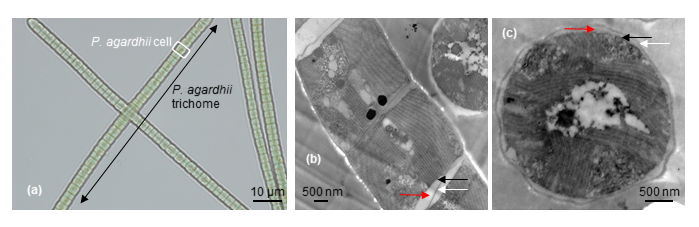

Supplement: S1 Fig — The red, white, and black arrows point at the peptiloglycan layer, the outer membrane, and cytoplasmic membrane, respectively. Photos: Chakib Djediat. (TIF) [file pone.0222029.s001.tif]

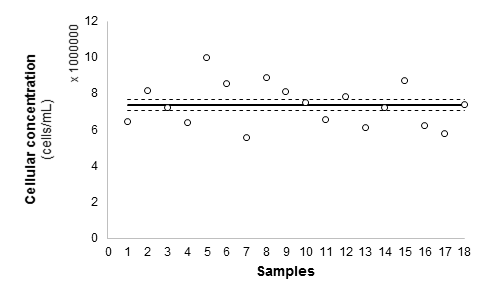

Supplement: S2 Fig — The solid line represents the mean cellular concentration (n = 18), (± SD; dashed lines). (TIF) [file pone.0222029.s002.tif]

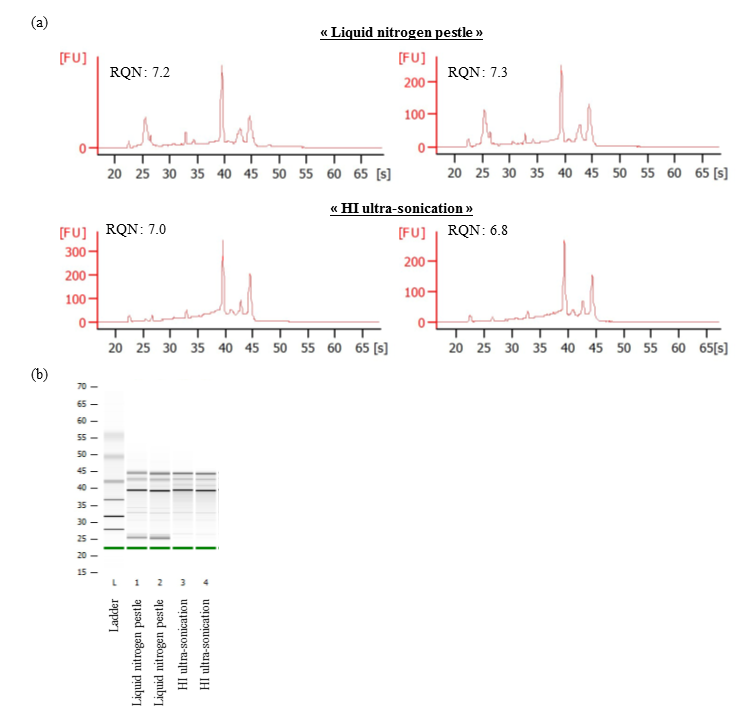

Supplement: S3 Fig — (a) 1: RNA ladder; 2–3: Tween 95; 4–5: Triton X 95; 6–7: Tween 65; 8–9: Triton X and (b) 1: RNA ladder; 2–3: Liquid nitrogen pestle; 5–6: HI ultra-sonication. (TIF) [file pone.0222029.s003.tif]

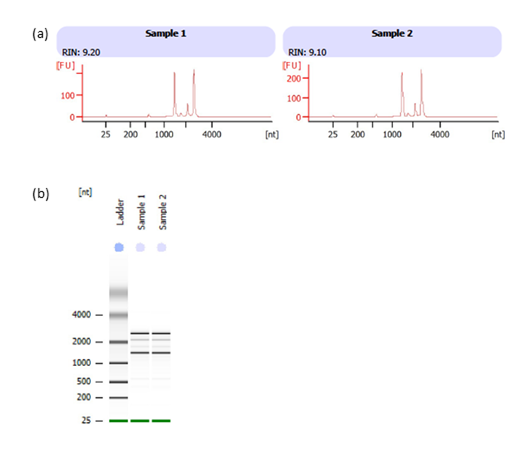

Supplement: S4 Fig — (TIF) [file pone.0222029.s004.tif]
